# Blood Group Determination using DNA extracted from Exfoliated Primary Teeth at Various Time Durations and Temperatures: A PCR Study

**DOI:** 10.5005/jp-journals-10005-1383

**Published:** 2016-12-05

**Authors:** Reshma K Pai, Sham S Bhat, Afreen Salman, Sundeep Hegde

**Affiliations:** 1 Reader, Department of Pedodontics and Preventive Dentistry Yenepoya Dental College, Mangaluru, Karnataka, India; 2 Professor and Head, Department of Pedodontics and Preventive Dentistry Yenepoya Dental College, Mangaluru, Karnataka, India; 3 Senior Lecturer, Department of Pedodontics and Preventive Dentistry Yenepoya Dental College, Mangaluru, Karnataka, India; 4 Professor and Head, Department of Pedodontics and Preventive Dentistry Yenepoya Dental College, Mangaluru, Karnataka, India

**Keywords:** ABO, Blood grouping, Human identification, Polymerase chain reaction, Primary tooth, Pulp.

## Abstract

**Aim:**

To determine polymerase chain reaction (PCR)-based blood group on tooth pulp obtained from teeth stored for 1 month, 6 months, and 1 year following extraction and to evaluate the stability of deoxyribonucleic acid (DNA) in primary tooth subjected to a temperature of 200°C ± 5°C for 15 minutes.

**Materials and methods:**

Dental pulp tissue was collected from 40 exfoliated primary teeth stored for various time durations and temperature and preserved at 4°C till DNA extraction was carried out. Deoxyribonucleic acid was extracted using silica membrane-based spin-column procedure of QIAamp DNA minikit from BioRad. Deoxyribonucleic acid was subjected to PCR amplification and monoplex allele-specific PCR primers for ABO genotyping.

**Statistical analysis used:**

The data were analyzed by comparison (based on percentage).

**Results:**

In our study, overall, 85% samples showed a DNA yield. Cent percent results were obtained for samples studied at the end of 1 month followed by 90 and 80% for samples studied for 6 months and 1 year respectively. Heated samples showed 70% result.

**Conclusion:**

Polymerase chain reaction was found to be an effective method for blood group determination for teeth stored at various time durations and temperatures. However, as the time interval increased, the number of positive results obtained decreased.

**How to cite this article:**

Pai RK, Bhat SS, Salman A, Hegde S. Blood Group Determination using DNA extracted from Exfoliated Primary Teeth at Various Time Durations and Temperatures: A PCR Study. Int J Clin Pediatr Dent 2016;9(4):308-312.

## INTRODUCTION

Teeth are the most durable and hardest of all tissues in the body and can survive long after soft and skeletal tissues have been destroyed and can be preserved intact for long periods of time after death.^1^ They are stable chemically, and they retain their characteristics even in the most adverse conditions.^2^ Despite the exposure of the body to any type of injury, it is usually possible to extract deoxyribonucleic acid (DNA) in sufficient quantity from pulp tissue, and this can be used as an aid in the identification of individuals.^3^

Identification connotes “determination or establishment of individuality of person - living or dead.”^4,5^ The identification of an unknown individual has always been of paramount importance to society. Blood group has been one of the cornerstones for identifying the biological materials in forensic investigations.^6^ Forensic odontology is an investigative aspect of dentistry that analyzes dental evidence for human identification.^7^

Lattes has aptly said, “The fact that belonging to a definite blood group is a fixed character of every human being and can be altered neither by the lapse of time nor by the intercurrent disease.” The presence of blood group substances and other genetic material like DNA in soft and hard dental tissues makes it possible to assist in the identification of highly decomposed bodies.^8^

Pulp tissue is one of the most protected of oral tissues, being surrounded on all the sides by dental hard tissues. Despite any depth of injury to the body, it is possible to extract DNA from the pulp tissue of the tooth.^4^

In the present-day scenario where the number of crimes against children in the form of battering, physical/ sexual abuse, kidnapping, and abduction is on the rise, exfoliated primary teeth may be the only source of evidence available at the crime scene. The dental pulp available from a primary tooth even though in minor quantity can prove to be extremely useful if standardized and advanced methods of DNA analysis are used.

Blood group determination can be done from the pulp tissue by using methods like absorption elusion, hemoagglutination, histochemical technique . Polymerase chain reaction (PCR) stands above all the mentioned methods with high rate of sensitivity and specificity.^2^ Deoxyribonucleic acid analysis technique, PCR, can be done even from the specimens of limited amount and partially degraded DNA as well and is fast, sensitive, and one of the most reliable analyses in the forensic field.^9^

Hence, this study was undertaken to determine the blood group from the exfoliated/extracted primary teeth of children using PCR.

**Fig. 1:**
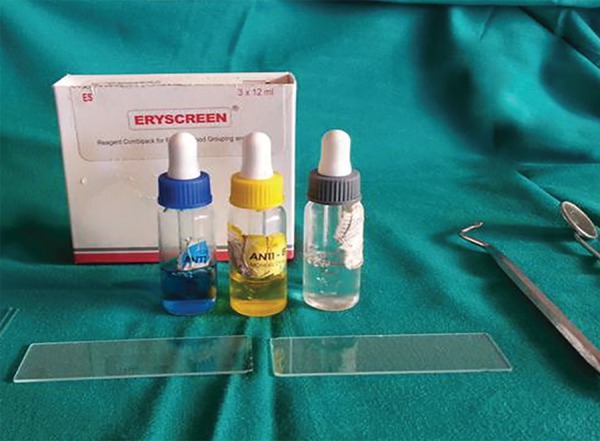
ABO blood grouping by slide agglutination method

**Fig. 2:**
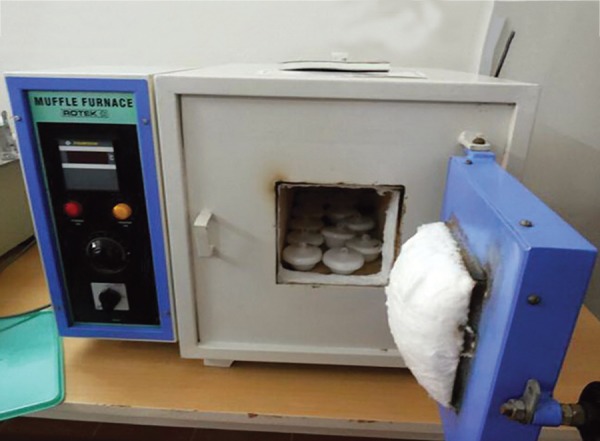
Heating of samples in muffle furnace

## OBJECTIVES

- Blood group determination by slide agglutination on fresh blood collected from socket following the extraction of primary tooth.
- PCR-based blood group determination on tooth pulp obtained from teeth stored for 1 month, 6 months, and 1 year following extraction.
- To evaluate the stability of DNA in primary tooth subjected to a temperature of 200°C for 15 minutes.
- To evaluate the application/usefulness of PCR as a tool for blood group determination from exfoliated/ extracted primary teeth in children.

## MATERIALS AND METHODS

### Sample Collection

A total of 40 exfoliated/extracted primary teeth for the study were collected from the children of age group 6 to 13 years reporting to the Department of Pedodontics and Preventive Dentistry, after obtaining ethical clearance from the ethical board of Yenepoya University and written informed consent from parents.

### Exclusion Criteria

Root canal-treated teeth, grossly decayed and infected teeth.

### Inclusion Criteria

Primary teeth with physiologic mobility or those indicated for serial extraction. Following routine tooth extraction procedure, blood was collected from the freshly extracted socket, and blood group was determined by slide agglutination method, and this was used as the control in the study (Fig. 1).

### Storage of Teeth

Extracted teeth were stored in saline at room temperature for various time durations.

### Grouping

Samples were randomly divided into four groups with 10 teeth in each group.

Group A - Teeth studied at the end of 1 month

Group B - Teeth studied after 6 months

Group C - Teeth studied after 1 year

**Fig. 3:**
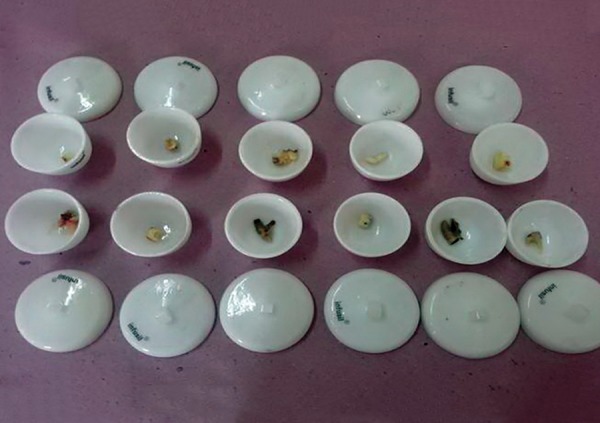
Heated samples

Group D - Teeth studied after subjecting to temperature of 200°C ± 5°C for 15 minutes (Figs 2 and 3).

### Collection and Storage of Dental Pulp Tissue

**Fig. 4:**
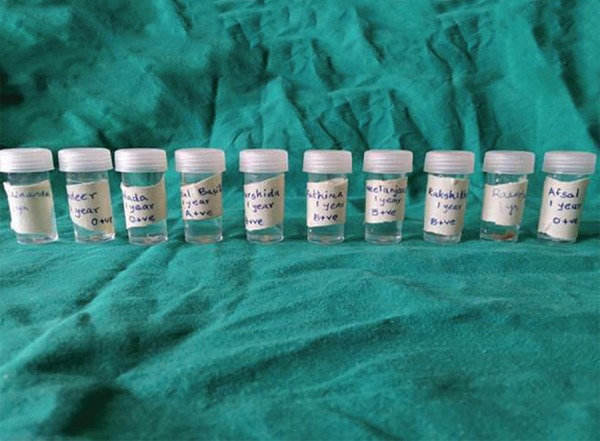
Pulp tissue stored in saline

Access opening was done on the collected teeth samples, and dental pulp tissue was collected from each sample using a barbed broach, which was then placed in vials with sterile normal saline and preserved at 4°C till DNA extraction was carried out (Fig. 4).

**Fig. 5:**
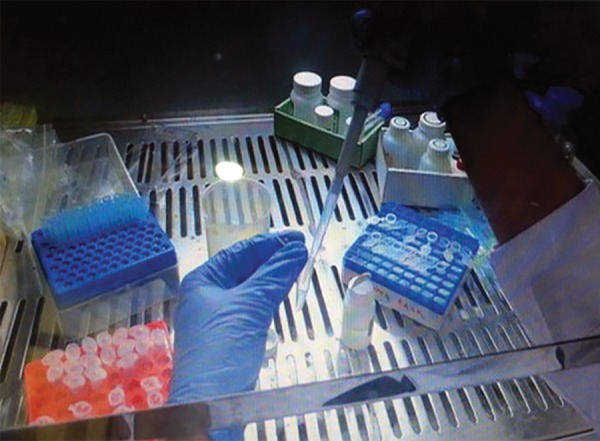
DNA extraction

**Fig. 6:**
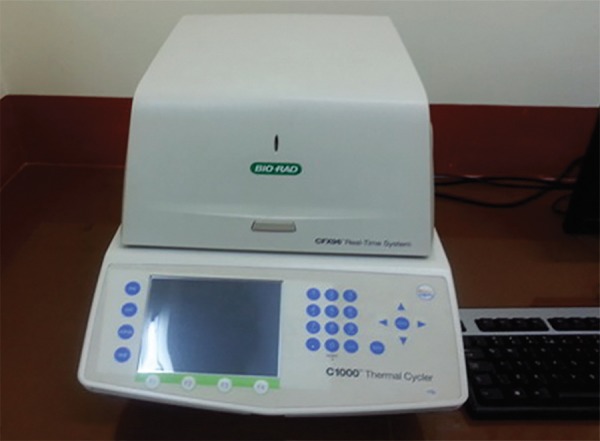
BioRad

### Deoxyribonucleic Acid Extraction

Pulp tissue was initially digested by Proteinase K, and DNA was extracted using silica membrane-based spin-column procedure of QIAamp DNA minikit from Bio-Rad (Figs 5 and 6) and monoplex allele-specific PCR primers for ABO genotyping.

Following are the primers that were used.^10^

Primer                    Sequence (5’-3’)

ABO261d-F          AGGAAGGATGTCCTCGTGTTAC

ABO261-R           GTTCTGGAGCCTGAACTGCT

ABO526C-F          AGCTGTCAGTGCTGGAGATGC

ABO526-R            TCCACGCACACCAGGTAATC

ABO803G-R         CCGACCCCCCGAAGTACC

ABO803-F            GAGATCCTGACTCCGCTGTT

Each sample was tested separately for the presence of blood groups A and B antigens. Absence of both the antigens in a sample indicated blood group O. Presence of both blood groups A and B antigens was seen in blood group AB. Amplification and analysis of amplified DNA products were performed using Biorad CFX 96 real-time PCR detection system. After an initial denaturation at 95°C for 10 minutes, amplification was performed by using 35 cycles of 95°C for 5 seconds, annealing at 65°C for 10 seconds, and extension at 72°C for 1 minute. Final reaction mixture of 25 μL per sample contained 15 μL of master mix containing cyber green, 2 μL of primers, and 8 μL of extracted DNA.

The results were tabulated. The data were analyzed by comparison (based on percentage).

## RESULTS

In our study, overall, 85% samples showed a DNA yield (Table 1). All the samples studied at the end of 1 month yielded DNA with 100% result (Table 2), 90% at the end of 6 months (Table 3) , 80% at the end of 1 year (Table 4), and heated samples showed 70% result, which was amplified and blood group identified by using real-time PCR (Table 5).

**Table 1:** **Table 1:** Overall percentage of blood grouping for all samples

		*Control group*				*Study group*			
*Total samples*		Positive*		*Negative*		Positive*		*Negative*	
		40 (100%)*		0(0%)		34 (85%)*		6 (15%)	
Total positive		85%*				15%*			

**Table 2:** **Table 2:** Blood grouping 1 month after extraction

		*Control group*				*Study group*			
*Blood group*		Positive*		*Negative*		Positive*		*Negative*	
A		2 (20%)*		0 (0%)		2 (20%)*		0 (0%)	
B		4 (40%)*		0 (0%)		4 (40%)*		0 (0%)	
AB		0 (0%)*		0 (0%)		0 (0%)*		0 (0%)	
O		4 (40%)*		0 (0%)		4 (40%)*		0 (0%)	
Total positive		10 (100%)*		0 (0%)		10 (100%)*		0 (0%)	
		10 (100%)*				10 (100%)*			

Results were considered positive for those samples whose results obtained by PCR coincided with the blood grouping performed by slide agglutination method on fresh blood from the tooth socket.

**Table 3:** **Table 3:** Blood grouping 6 months after extraction

		*Control group*				*Study group*			
*Blood group*		Positive*		*Negative*		Positive*		*Negative*	
A		5 (50%)*		0 (0%)		4 (40%)*		1(10%)	
B		3 (30%)*		0 (0%)		3 (30%)*		0 (0%)	
AB		1 (10%)*		0 (0%)		1 (10%)*		0 (0%)	
O		1 (10%)*		0 (0%)		1 (10%)*		0 (0%)	
Total positive		10 (100%)*		0 (0%)		9 (90%)*		1(10%)	
		10 (100%)*				9 (90%)*			

**Table 4:** **Table 4:** Blood grouping 1 year after extraction

		*Control group*				*Study group*			
*Blood group*		Positive*		*Negative*		Positive*		*Negative*	
A		2 (20%)*		0 (0%)		1 (10%)*		1 (10%)	
B		5 (50%)*		0 (0%)		4 (40%)*		1 (10%)	
AB		0 (0%)*		0 (0%)		0 (0%)*		0 (0%)	
O		3 (30%)*		0 (0%)		3 (30%)*		0 (0%)	
Total positive		10 (100%)*		0 (0%)		8 (80%)*		2 (20%)	
		10 (100%)*				8 (80%)*			

**Table 5:** **Table 5:** Blood grouping from heated samples after extraction

		*Control group*				*Study group*			
*Blood group*		Positive*		*Negative*		Positive*		*Negative*	
A		1 (10%)*		0 (0%)		1 (10%)*		0 (0%)	
B		2 (20%)*		0 (0%)		1 (10%)*		1 (10%)	
AB		1 (10%)*		0 (0%)		1 (10%)*		0 (0%)	
O		6 (60%)*		0 (0%)		4 (40%)*		2 (20%)	
Total positive		10 (100%)*		0 (0%)		7 (70%)*		3 (30%)	
		10 (100%)*				7 (70%)*			

## DISCUSSION

Forensic identification has evolved into an art of science and involves various specialties. As teeth play an important role in forensic field, extracted DNA from the pulp tissue of the tooth can be used to discriminate one individual from another.

Blood group determination from teeth using the PCR analysis can provide an important means of personal identification in the event of mass disaster, such as airplane crash or fire.

Although there are various blood grouping techniques, PCR is the most standardized technique in forensic field since a high rate of sensitivity and specificity has been noted in previous experiments using samples like saliva, blood, and semen.^2^

The use of blood group antigens in medicolegal examination is based on the fact that once a blood group is established in an individual, it remains unchanged throughout his/her life. The presence of blood group antigens in dental tissues makes it possible to assist in identifying highly decomposed bodies where teeth and bone are the only significant tissues remaining (Xingzi et al).

The dental pulp undergoes degeneration, necrosis, and putrefaction inside an exfoliated tooth, which takes a period of weeks to months.^9^ Thus, checking the usefulness of the pulpal remains during and after a period of time lapse was necessary. Hence, in our study, we used the teeth specimens stored at an interval of 1 month, 6 months, and 1 year after extraction and after heating (200°C ± 5°C for 15 minutes).

Determination of blood group was done using pulp and correlated with the blood grouping of blood collected from the extraction socket of the same subject. In our study, 34 teeth out of 40 showed positive results, with 85% success using pulp by PCR, and control group showed 100% results. It was observed that as the time interval increased, the number of positive results obtained decreased (1 month - 100%, 1 year - 80%). The overall decrease in the success rate could be due to contamination of the tooth, time lapse for the procedure, variation in the pulp volume, loss of tissue during pulp extirpation, and root resorption in the deciduous tooth.^11^

In a study conducted by Inamdar et al, in the blood group determination using absorption elution technique from exfoliated primary teeth, 40% teeth showed positive result on the 30th day, 20% on the 90th day, and 0% on the 180th day.^12^

In the present study, it was possible to extract DNA from the pulp tissue from 7 out of the 10 heated samples, which were subjected to a temperature of 200°C ± 5°C, with 70% success rate. Tsuchimochi et al conducted a study to extract DNA from dental pulp for PCR analysis where the extracted teeth were subjected to temperatures of 100, 200, 300, 400, and 500°C for 2 minutes. All samples heated up to 300°C could be amplified, whereas those subjected to temperatures above 400°C did not produce any PCR product.^13^

Korszun et al determined the thermostability of ABO blood group antigens in human dental pulp and stated that dentin and enamel are poor insulators and give inadequate thermal protection to pulp when the external temperature rises 200°C or more. Therefore, at higher temperatures, only those teeth protected from tongue, cheeks, or bone would be expected to exhibit ABO antigens.^14^

Evaluation of the PCR assay in comparison to slide agglutination test has enabled us to assess the accuracy and usefulness of this test with a good success rate. Thus, our study emphasizes that the blood grouping of tooth pulp by PCR method can be used for relative identification of individuals, which will be of immense value to forensic dentistry.

## CONCLUSION

As the results of our study are encouraging with an overall success rate of 85%, blood group determination from teeth using the PCR analysis can provide an important means of personal identification in the event of mass disaster, such as airplane crash or fire. Hence, a doctor’s role as forensic pathologist and forensic odontologist goes hand in hand with the police officer in establishing the identity of an individual in mass disaster. Once this technique is standardized using a larger sample size and various other environmental conditions in determining the blood group from pulp tissue, these benefits can be offered to the society when need arises.
